# Unusual adsorption behaviours and responsive structural dynamics *via* selective gate effects of an hourglass porous metal–organic framework†

**DOI:** 10.1039/c9ra07301a

**Published:** 2019-11-14

**Authors:** Ying Xiong, Yan-Zhong Fan, Zhang-Wen Wei, Cheng-Xia Chen, Sha Chen, Dawei Wang, Mihail Barboiu, Ji-Jun Jiang, Cheng-Yong Su

**Affiliations:** MOE Laboratory of Bioinorganic and Synthetic Chemistry, Lehn Institute of Functional Materials, School of Chemistry, Sun Yat-Sen University Guangzhou 510275 P. R. China jiangjij@mail.sysu.edu.cn; Life Science Institute, Jinzhou Medical University Jinzhou 121001 P. R. China; Adaptive Supramolecular Nanosystems Group, Institut Européen, des Membranes Place Eugène Bataillon CC047 34095 Montpellier Cedex 5 France; State Key Laboratory of Applied Organic Chemistry Lanzhou University Lanzhou 730000 P. R. China

## Abstract

An hourglass porous metal–organic framework, LIFM-12, constructed on a T-shaped flexible ligand with Cu^2+^ paddle-wheel clusters, shows temperature and gas adsorption responsive structural dynamics upon reversible molecular guest binding. Temperature-dependent single crystal and powder X-ray diffraction experiments show that the open gate status of the framework with adaptive behaviours facilitates kinetic diffusion of gas molecules resulting in the sequential filling of pores of different sizes, thus creating a breathing behaviour reminiscent of the observation of several steps in adsorption isotherms. In addition, adsorption studies revealed that LIFM-12 performs exceptional adsorption selectivity of 10–25 for CO_2_*versus* light gases N_2_, CH_4_, and CO and up to 200 for C_3_H_6_*versus* CH_4_.

## Abstract 2

A Cu^2+^ based metal–organic framework exhibits dynamic behaviour upon guest inclusion/release process, and performing stepwise sorption isotherms for various gas. We elucidate detailed mechanisms under its exceptional sorption behavior.

## Graphical abstract

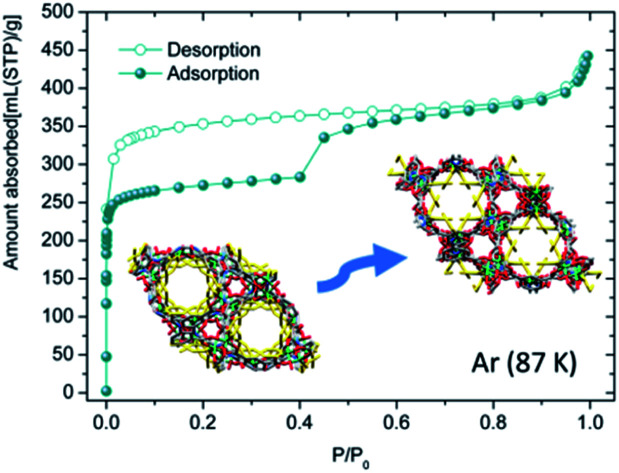

## Introduction

Metal–organic frameworks (MOFs) is a hot research topic in the past decades and attracts intense interest in the investigation of their fascinating structures and potential applications such as separation,^1–4^ gas storage,^5–8^ and catalysis.^9–13^ Many efforts focus on structural design, surface modification and functional tuning of the rigid coordination frameworks. On the other hand, it has been found that some unique properties, like extraordinary gas adsorption/separation behaviors, can be obtained *via* internal structural dynamics. For example, gate-opening^14–16^ and breathing^17–20^ behaviors are important known phenomena, occurring through reversible structural transitions between two or more states triggered *via* pressure or temperature variations or guest binding, providing a unique approach to develop multifunctional porous materials.^21,22^

The dynamic behaviors of MOFs in response to external stimuli, can be triggered by several mechanisms, such as sliding of interlocked fragments of interpenetrated frameworks,^23,24^ coordination geometry rearrangements of metal centers or clusters,^25–27^ rotational motion involving flexible parts of bridging ligands,^16,28–33^ or a combined mechanism containing more than one of the above three. Since interpenetration often leads to a shrink of solvent accessible volume,^34–36^ we pay more attention to the geometry rearrangement of the coordination environment and to the bond rotation of organic linkers.^20,37–41^ If the framework flexibility and dynamics are generated by the bond rotation, it usually neither changes the topology of the whole framework nor dramatically reduces porosity. Moreover, it can temporarily open the narrow apertures of the coordination framework to facilitate kinetic diffusion of guest molecules and/or accept oversized guest molecules to give stepwise sorption behavior,^42–44^ thus providing specific applications for gas separation, bio-chemical sensing, *etc.* Although the dynamic behaviors have been discovered in lots of MOFs and fruitful research has been conducted, the detailed interpretation of the mechanisms of such behaviors, especially from the aspects of crystalline structures by X-ray single crystal diffraction, is still rare,^45,46^ partly because MOFs may easily lose its crystalline when they are under heating (or cooling) and/or guest inclusion (or exclusion) process. In addition, comprehensive studies upon the temperature and guest effects on the dynamic structures are also highly required.

**Scheme 1:**
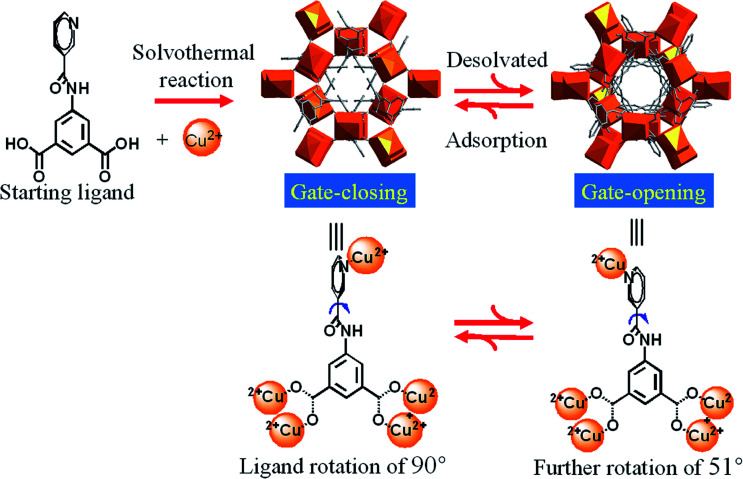
Gate opening/closing *via* guest removal and inclusion processes, controlled by the dynamic rotation of pyridyl ring and amide group of H_2_NIA ligand.

In this paper, we report an hourglass porous MOF constructed from a T-shaped flexible ligand and Cu^2+^ paddle-wheel clusters, behaving variable gate effects, structural dynamics and excellent framework robustness induced by covalent bond rotation (Scheme 1) in response to temperature and gas inclusion/release, without loss of the single crystallinity. Experimental single-crystal X-ray diffraction, X-ray powder diffraction (PXRD), gas adsorption and theoretical simulations have been combined to elucidate the detailed structural dynamics for the stepwise adsorption isotherms, disclosing exceptional adsorption selectivity of 10–25 for CO_2_*versus* light gases N_2_, CH_4_ and CO and up to 200 for C_3_H_6_*versus* CH_4_ hydrocarbons.

## Results and discussion

### Solid crystal structure of LIFM-12

Solvothermal reactions of Cu^2+^ with similar T-shaped ligands 4-pyridylaminocarbonyl (H_2_INIA) and 1-oxidopyridin-1-ium-4-ylamino carbonyl (H_2_INOIA) afforded two **rtl** topological Cu-MOFs, LIFM-10(Cu), LIFM-11(Cu) through a ligand-to-axial pillaring strategy.^47–49^ MOF of [Cu(C_14_N_2_O_5_H_8_)]·1.33DMF·2H_2_O (LIFM-12) was constructed through a similar strategy with the modified ligand H_2_NIA (Table S1†). The T-shaped ligand, 5-(nicotinoylamino)isophthalic acid (H_2_NIA), prepared for the studies described here, contains two carboxylates and a pyridine coordinating sites, as well as an amido group that can potentially bind the guests and serves as a rotating engine under external stimulus (Scheme 1). Single crystals of LIFM-12 were obtained by solvothermal conditions.

**Fig. 1:**
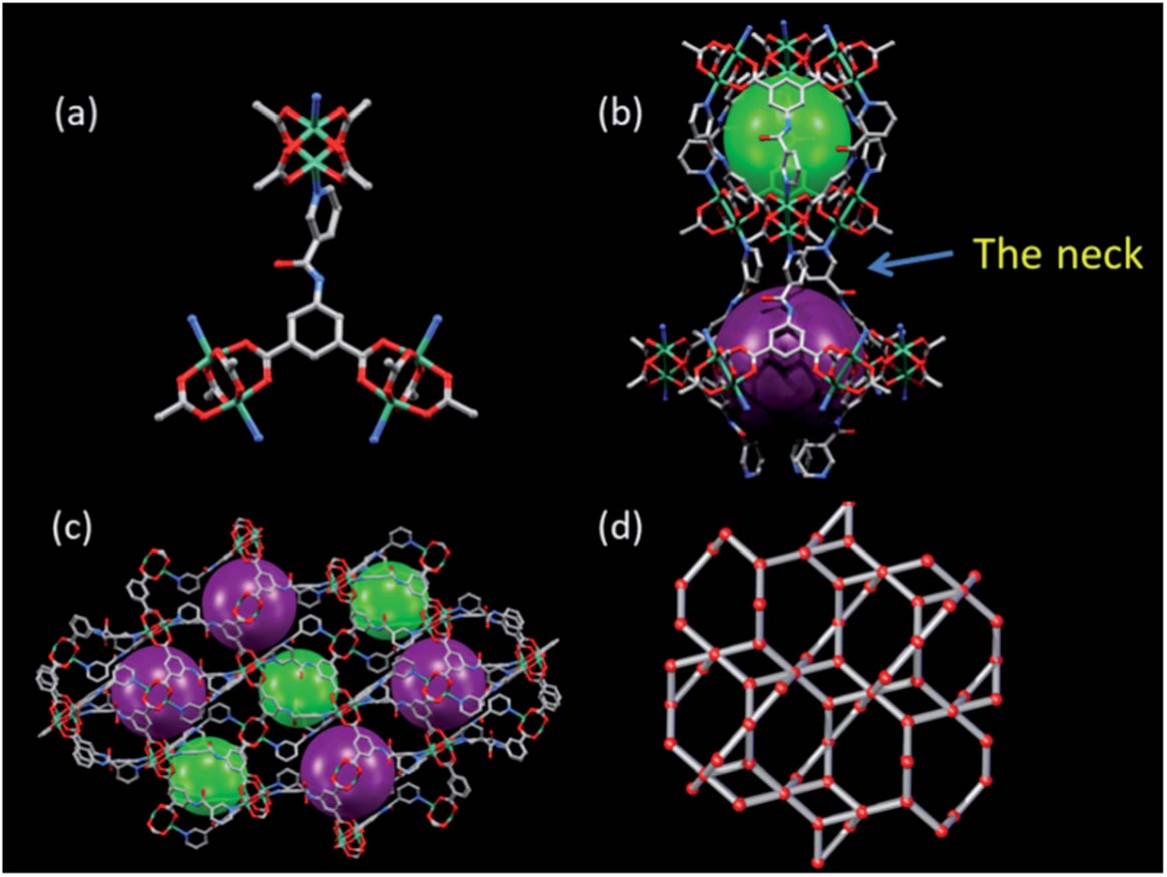
(a) The ligand NIA^2−^ and the Cu^2+^ paddle wheel units of LIFM-12, (b) cavities A and B, connected *via* a narrow neck within the structure of MOF (c) crystal packing and (d) topological representations of the porous framework of LIFM-12.

The backbone is composed of paddle wheel binuclear Cu_2_-units, bridged by the organic linker NIA^2−^ (Fig. 1a) to generate 3D microporous architecture (Selected bond length and angles are listed in Table S2†). A one-dimensional (1D) hourglass channel is formed two cavities of different sizes alternately aligned along *c*-axis: the small Cavity A is about 10 Å in diameter and looks like a Chinese drum (Fig. 1b), while the larger Cavity B is about 12 Å in diameter and shows turbinate shape (Fig. 1c). The cavities A and B are connected through a narrow neck constituted of three pyridyl rings (Fig. 1b). Therefore, although LIFM-12 exhibits the same **rtl** topology^50–52^ (Fig. 1e and S1†) as LIFM-10(Cu), the porous nature is completely different. When comparing two structures, we can find that the ligand INIA^2−^ in LIFM-10(Cu) is co-planar but displays a slight bending shape to satisfy the connection requirement of **rtl** topology (Fig. S2a†), suffering some framework stress to hinder the amide group from rotation. While in LIFM-12, the pyridyl ring and amide group of NIA^2−^ rotate from the basic benzene plane to coordinate with Cu^2+^, fulfilling the requirement of **rtl** topology without bending stress (Fig. S2b†). The rotation of the pyridyl groups inward to the pores leads to a narrow junction between cavities A and B, thus controlling the gas diffusion along the 1D hourglass channel (*vide infra*). PLATON calculation indicates that potential solvent accessible void of LIFM-12 framework is 51.4% (all solvent molecules are ignored to calculate the potential solvent accessible void, *sic passim*).^53^

### Stability of LIFM-12

**Fig. 2:**
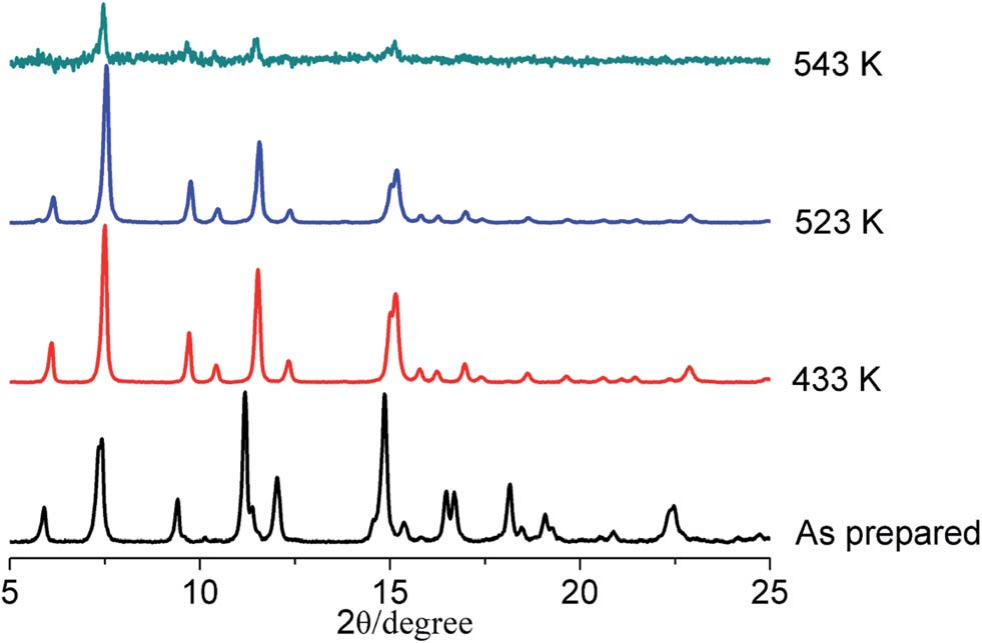
Temperature-dependent PXRD patterns of LIFM-12.

Thermal gravimetric analysis (TGA) shows that the framework of LIFM-12 is stable upon heating up to 531 K under N_2_. The weight-loss ratio before 531 K is 21.5%, corresponding to 1.33 DMF and 2 water molecules for each asymmetric unit (Fig. S4†). The phase purity for LIFM-12 is identified by PXRD (Fig. S5†). Bulk samples are stable in air for half a year and maintain the porous structure after being desolvated at 433 K under a dynamic vacuum at 10^−3^ torr for 20 hours (Fig. S6†). It is not stable after immersing in water after a week (Fig. S7†). Interestingly, LIFM-12 is stable in a selected solvent like methanol, chloroform, and tetrahydrofuran at room temperature (Fig. S8†). Temperature-dependent PXRD measurements have been carried out under the N_2_ atmosphere for LIFM-12. As shown in Fig. 2 and S9,† the framework can persist its backbone around 523 K after removal of the solvent in the lattice. Peaks shifted a little at 2*θ* = 7.4° and between 10.9–12.3° and 14.2–15.7° which should be attributed to slight readjustment during the heating process.

### Structural dynamics upon heating

**Scheme 2:**
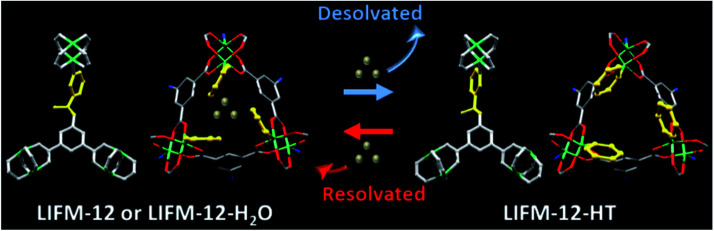
Desolvation/resolvation processes of LIFM-12. Water molecules in LIFM-12 or LIFM-12-H_2_O are represented by silver balls.

Temperature-dependent PXRD of LIFM-12 analysis showed a robust framework upon heating at high temperature and then cooling under air and N_2_. Three new X-ray single crystal structures of LIFM-12-HT (heated at 435 K under N_2_), LIFM-12-LT (heated at 435 K then cooling down to room temperature under N_2_) and LIFM-12-H_2_O (heated at 435 K then cooling down under air to room temperature) were afforded in this *in situ* process. LIFM-12-HT showed a decline of the cell volume of 4.3% and a slight shortening of three axes of cell parameters. The potential solvent accessible voids reduced from 51.4% to 45.7% as calculated by Platon.^54^ No guest molecules are observed in the apertures. The desolvated framework reveals that the amide group and terminal pyridyl rings rotate slightly after removal of guest molecules outside the channels, the rotational angle of the amide groups is 50.8° (Fig. S10†). The most striking difference is that the neck between cavities A and B turns open by the rotation of amide and pyridine groups (Scheme 2). This state was named as open pore model (OPM). Crystal volume and potential solvent accessible voids of LIFM-12-LT remain almost the same, giving unchanged chemical content (Table S3†). LIFM-12-LT can reabsorb water molecules when exposed to air.

Then following water re-adsorption, the hydrated framework is similar with LIFM-12 while the amide and pyridine groups switch back to partly shield the neck between cavities A and B. The potential solvent accessible void slightly expands from 46.1% to 52.7% and is similar with the void of primitive LIFM-12 (Fig. S11†). This new structure form was named as closed pore model (CPM). The above results conclude that the framework of LIFM-12 is overall robust but the organic ligands exhibit mobility upon heating/cooling, which is in agreement with the peak shift at 2*θ* = 7.4° and between 10.9–12.3° and 14.2–15.7° of VT-PXRD patterns (Fig. S12†).

### Gas adsorption and selectivity

**Fig. 3:**
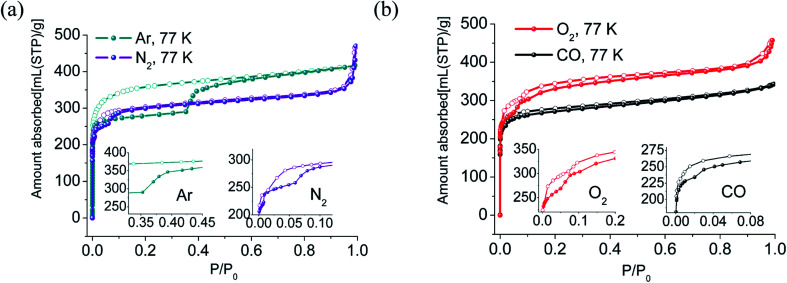
Gas sorption isotherms of N_2_, Ar (a) O_2_, CO (b) measured at 77 K. Solid symbols: adsorption, open symbols: desorption. Inset: curves of partial enlargement of corresponding sorption isotherms (Saturation pressure at 77 K of Ar: 210 torr; N_2_: 760 torr; O_2_: 156 torr; Ar: 453 torr).

The N_2_, O_2_, CO, Ar adsorption isotherms of activated sample LIFM-12 were performed at 77 K. The N_2_ sorption isotherms display similar Type-I sorption behavior. Interestingly, the adsorption branch of isotherms exhibits three distinct steps in the relative pressure range of 10^−7^∼0.007, 0.007–0.06 and 0.06–0.90. The BET surface area calculated in the range of *P*/*P*_0_ = 0.069–0.089 is 1221.1 m^2^ g^−1^, the pore volume is estimated to be 0.51 cm^3^ g^−1^ at *P*/*P*_0_ of 0.90 (Table S4†). The O_2_ adsorption isotherms exhibit three distinct steps ending at *P*/*P*_0_ = 0.05, 0.1 and 0.3, respectively. The adsorption isotherms of CO and Ar at 77 K present two steps, with the second step beginning at *P*/*P*_0_ = 0.02 for CO and at 0.35 for Ar (Table 1). Interestingly, large hysteresis that closes to the adsorption branches at very low pressure in each isotherm were observed. They can be attributed to the trapping effect of the gas in the pores^53^ caused by the disadvantaged diffusion at a rather low temperature (Fig. 3).

**Table 1:** The regions of steps for each gas at 77 K

	The region of the first step (*P*/*P*_0_)	The region of the second step (*P*/*P*_0_)	The region of the third step (*P*/*P*_0_)	Hysteresis
CO	∼0.02	0.02–	—	○
Ar	∼0.35	0.35–	—	○
O_2_	∼0.05	0.05–0.10	0.1–	○
N_2_	∼0.007	0.007–0.06	0.06–	○

**Fig. 4:**
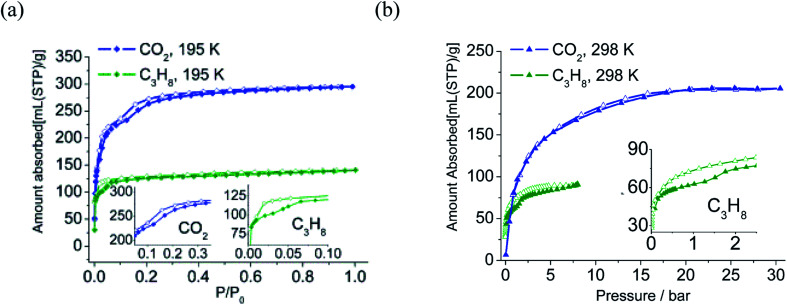
Gas sorption isotherms of CO_2_, C_3_H_8_ measured at 195 K (a) and 298 K (b) solid symbols: adsorption, open symbols: desorption. Inset: curves of partial enlargement of corresponding sorption isotherms at high pressure and 298 K.

As viewed in Fig. 4, both the CO_2_ and C_3_H_8_ adsorption isotherms at 195 K show two distinct steps, and the desorption branch of C_3_H_8_ shows clear hysteresis. CO_2_ adsorption at 298 K and 30 bar indicates none of the steps. The C_3_H_8_ adsorption isotherm at 298 K exhibits stepwise behaviors and hysteresis. The vapor adsorption isotherms of methanol, ethanol, and propanol all exhibit two-step stepwise behavior. The total adsorption amount decreases as critical molecular size increases following the sequence of MeOH > EtOH > *n*-PrOH (Fig. S13†).

Low pressure CO_2_ adsorption analysis at 273 K, 298 K and 308 K achieved 125.6, 87.0, 56.1 ml g^−1^ (5.6, 3.9, 2.5 mmol), which belongs to commendable values among MOFs (Fig. S14 and Table S5†). The coverage-dependent adsorption isosteric heat (calculated based on the virial method^55–57^) lies in a range of 31.2–28.8 kJ mol^−1^ (Fig. S15†). Comparison with MOFs without UMCs (Unsaturated Metal Centers) shows that LIFM-12 possesses lower adsorption heat than that in CuBTTri-mmen (96 kJ mol^−1^),^58^ Bio-MOF-11 (47 kJ mol^−1^)^59^ and {[CuL]·DMF·2H_2_O}_*n*_ (46 kJ mol^−1^)^60^ and slightly surpasses Cu(bpy-1)_2_(SiF_6_) (27 kJ mol^−1^).^61^ Such low adsorption heat denotes that LIFM-12 does not have a high affinity effect with CO_2_, thus regeneration procedure for industrial applications can be the energy conservation (please note that the adsorbed amounts of CO_2_ (298 K) are lower than for CuBTTri-mmen (4.2 mmol g^−1^), Bio-MOF-11 (4.1 mmol g^−1^), {[CuL]·DMF·2H_2_O}_*n*_ (5.0 mmol g^−1^) or Cu(bpy-1)_2_(SiF_6_) (6.8 mmol g^−1^)).

**Fig. 5:**
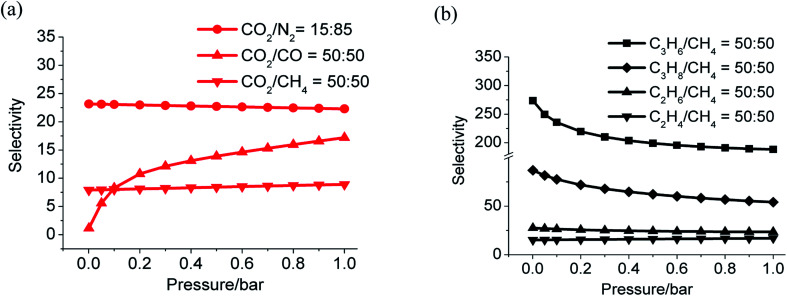
Gas selectivity calculated by IAST, CO_2_*versus* N_2_, CO, CH_4_ (a) and C_3_H_6_, C_3_H_8_, C_2_H_4_, C_2_H_6_*versus* CH_4_ (b) at 298 K.

Gas selectivities of CO_2_/N_2_, CO_2_/CH_4_, and CO_2_/CO at 298 K based on ideal adsorption solution theory (IAST)^62^ were determined using experimental isotherms of CO_2_, CH_4_, CO and N_2_ (Fig. S19†). The values locate in the range of 23.2 to 26.1 for CO_2_/N_2_ (15 : 85), 7.9 to 8.9 for CO_2_/CH_4_ (50 : 50) and 1.0 to 1.1 for CO_2_/CO (50 : 50) (Fig. 5a). All these values are close to that calculated by the Henry's law selectivity (Fig. S16 and Table S6†).

The adsorption of light alkanes and alkene of LIFM-12 clearly show that selectivities of C_3_H_8_/CH_4_, C_3_H_6_/CH_4_, C_2_H_6_/CH_4_, and C_3_H_8_/CH_4_ are from 86.8 to 56.1, 273.5 to 188.3, 27.5 to 23.5 and 15.2 to 17.0 respectively (Fig. 5b and S17†), which are also similar with the ones calculated by the Henry's law method (Table S7†). Both the adsorption amount and selectivity of LIFM-12 are higher than most of the MOFs reported for hydrocarbon separation,^63^ only lower than the MOF-74 series with UMCs,^64^ thus distinguishing LIFM-12 as an efficiency candidate of separation material of C_2_/C_3_ hydrocarbons from CH_4_ at the mild condition.

### Theoretical simulations of gas adsorption

To understand the dynamics of LIFM-12 in the gas adsorption process, theoretical simulations using the Atom Volumes & Surfaces tool of Material Studio have been performed on LIFM-12-HT (OPM) and LIFM-12 (CPM). The simulation of N_2_ adsorption at 77 K show that BET surface area derived from CPM is 1253.7 m^2^ g^−1^, extremely close to the experimental value of 1221.1 m^2^ g^−1^LIFM-12, which calculated by the pressure higher than 0.06 *P*/*P*_0_ (the last step), showing that the framework turns into CPM after filling N_2_ gas. The pore volume of the CPM calculated from N_2_ adsorption is estimated to be 0.51 cm^3^ g^−1^ at *P*/*P*_0_ of 0.90, which is larger than that of simulation value (0.46 cm^3^ g^−1^ at *P*/*P*_0_ of 0.90). The deviation may be contributed to the unideal morphology of crystals, which creates a hierarchical porous macrostructure in the synthesis process (Table 2).

**Table 2:** Simulated solvent surface areas calculated by LIFM-12 and LIFM-12-HT

Model	Gas	Solvent surface (m^2^ g^−1^)	Pore volume (cm^3^ g^−1^)
LIFM-12	Ar (87 K)	1342.7	0.47
	N_2_ (77 K)	1253.7	0.463
LIFM-12-HT	Ar (87 K)	913.8	0.39
	N_2_ (77 K)	851.6	0.37

### Gas-induced *in situ* temperature-dependent PRXD

**Fig. 6:**
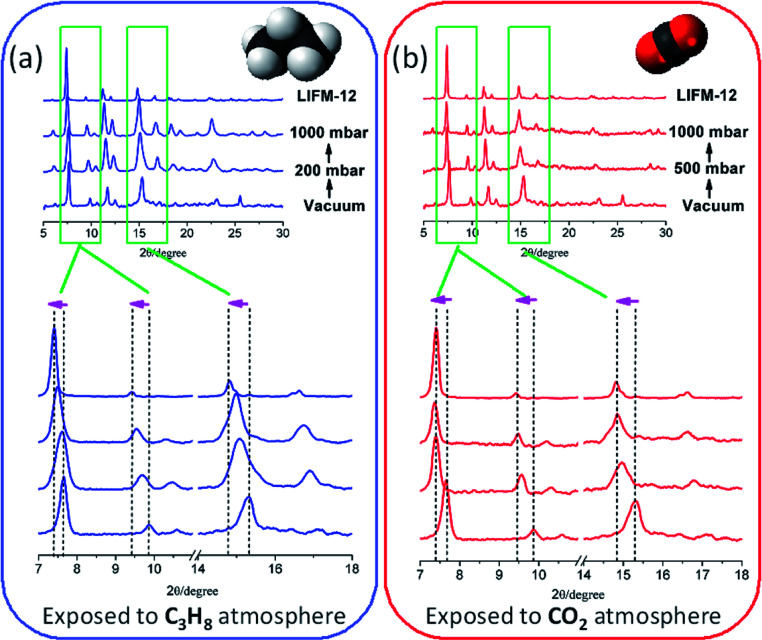
(a) C_3_H_8_ and (b) CO_2_ absorption and induced *in situ* related pressure-dependent powder X-ray analysis.

The dynamic structural variation of the framework in response to gas adsorption through bond rotation within NIA^2−^ ligand is interesting. To get more structural insights for this process, herein we continually perform experimental gas-induced *in situ* pressure-dependent powder X-ray analysis to reveal the structural changes of LIFM-12 by removing and refilling guests under controllable pressure (Fig. 6).

The measurements started from OPM under vacuum for each gas, then fill C_3_H_8_ into the chamber. The PXRD patterns show an obvious offset in which the peaks moving towards to low 2*θ* angles (Scheme 2a). By continuing to fill the gas to the pressure at 1000 mbar, the patters shift to even lower 2*θ* angles, approaching the patterns of CPM. The dynamic change from OPM to CPM by filling the gas can also be proved by the C_3_H_8_ adsorption isotherms. As shown in Fig. 6a, in the initial stage, the framework is OPM, the adsorption is still at the first step, by continuously filling the gas, it is speculated that the pressure increase from 300 to 1000 mbar, the imine bonds gradually rotate during the whole C_3_H_8_ adsorption process, finally end as the CPM when the second step of the adsorption completes (*P* > 2 bar).

The PXRD response to CO_2_ filling is carried out in a similar method. By introducing CO_2_ into the sample chamber to a pressure of 500 mbar, PXRD patterns immediately move to lower 2*θ* angles, then the patterns keep the same and are similar to the CPM without changes after CO_2_ filling to the pressure at 1000 mbar. The phase change upon CO_2_ adsorption may stem from small critical molecular size for CO_2_, and/or kinetically favourable for CO_2_ diffusion in apertures at room temperature (Scheme 2b).

The above results indicate that the gas filling dependent PXRD analysis is reminiscent with the multistep absorption process involving sequential filling of the pores of different sizes.^65^ Comparing to the reported results, herein the “gate-open” effect is different from the so-called one of MIL-53,^66^ which excludes guest molecules in gate-closed structure. The multi-stepwise gas adsorption behaviour of LIFM-12 can be ascribed of the partly accessible void between the cavities A and B, which can accommodate gases and exhibit framework flexibility, when the gate-open effect happens at specific pressure, the opened gate would kinetically facilitate adsorption process by relatively larger apertures, thus creating new steps in adsorption isotherms.

## Conclusions

In summary, a flexible MOF LIFM-12 has been constructed, in which its flexibility can be triggered by the rotating of imine bond under the heating process and gas pressure variation. Gas adsorption results reveal that it possesses noticeable gas uptake and gas selectivity ability. *In situ* related pressure-dependent PXRD verifies the stepwise adsorption starts from OPM, then develops to CPM by the increase of gas pressure variation. Our results provide a better understanding of the flexibility of MOFs upon the external stimulus and prop up the better design of responsive materials for particular applications in gas separation, molecular sensing, catalysis and *et al.* in the future.

## Experimental section

### Materials and physical measurements

All chemicals were obtained from commercial sources and used without further purification. Solid-state IR spectra were recorded using Nicolet/Nexus-670 FT-IR spectrometer in the region of 4000–400 cm^−1^ using KBr pellets. Mass spectra (MS) were recorded on a JEOL accuTOF-CS, JMS-T100CS mass spectrometer. Elemental analyses were performed by PerkinElmer 240 elemental analyzer. Powder X-ray diffraction (XRD) measurements were performed on a Bruker D8 ADVANCE diffractometer at 40 kV and 40 mA with a Cu target tube and a graphite monochromator. ^1^H NMR spectra were measured with a Varian Mercury Plus 300 MHz spectrometer. Thermogravimetric analysis (TGA) was performed on a NETZSCH TG209 system in nitrogen and under 1 atm of pressure at a heating rate of 10 K min^−1^.

***Synthesis of H_2_NIA*.** H_2_NIA ligand was prepared with a modified method as previously reported.^67^ A mixture of nicotinic acid (0.35 mol) and freshly distilled thionyl chloride (118 ml, 1.6 mol) were heated at 353 K for 6 hours. Then excess thionyl chloride was removed under flowing nitrogen gas. The resulting yellowish powder was used as prepared. Isonicotinoyl chloride hydrochloride in 20 ml dimethylacetamide (DMAc) was added dropwise to a solution of 5-aminoisophthalic acid (0.30 mol) and *N*,*N*-dimethylaminopyridine (0.10 mmol) in 80 ml DMAc at 273 K under nitrogen. Then the temperature was slowly raised to 313 K and kept at this temperature for 8 h. The cooled mixture was poured into water, a precipitate formed and was filtered off, washed with hot water twice and dried under vacuum at 373 K. The product was recrystallized twice in a mixture of dimethylformamide and water (DMF)/H_2_O (3/2). Yield: 70%.^1^H NMR [300 MHz, (CD_3_)_2_SO]: 10.76 (s, 1H), 9.13 (d, 1H), 8.76 (dd, 1H), 8.64 (d, 2H), 8.32 (tt, 1H), 8.21 (t, 1H), 7.58 (dd, 1H). MS [ESI^−^], *m*/z calc.: 285.06, found: 285.31.

***Synthesis of LIFM-12*.** H_2_NIA (0.1 mmol), Cu(NO_3_)_2_. (0.1 mmol) were mixed and dispersed in DMF (4.0 ml) and methanol (4.0 ml), then three drops of HNO_3_ was to this solution. The mixture was transferred into a 12 ml autoclave, which was sealed and heated to 393 K for 4000 minutes. After cooling over 12 hours to room temperature, blue, slice microcrystalline product was obtained, then washed with DMF/methanol (1/1) and dried in air. Yield: 43%. Elemental analysis (% calc./found): C: 45.10/44.82, H: 4.18/4.72, N: 9.73/9.72. Selected IR: *ν*/cm^−1^: 3435 (s), 1657 (s), 1639 (s), 1562 (s), 1477 (w), 1422 (s), 1375 (vs), 1293 (m), 1200 (w), 1111 (m), 1052 (m), 778 (m), 730 (m), 631 (w), 599 (w), 565 (w).

### Temperature-dependent X-ray single crystal study

A single crystal of LIFM-12 was carefully picked and coated in epoxy resin AB glue, attached to a glass silk inserted in a stainless steel stick, then quickly transferred to an Oxford Gemini S Ultra CCD diffractometer (Cu Kα radiation). Four sets of data were collected in sequence. (1) The single crystal exposed in a stream of nitrogen at 150 K. (2) The single crystal was slowly heated to 435 K in nitrogen, kept still for 1.5 hours, and then the data was collected in nitrogen. (3) The single crystal was slowly cooled to 298 K in nitrogen, held for 1 hour more, and then the data was collected in nitrogen. (4) The nitrogen stream was shut off and the single crystal was exposed to air for 1 hour more, then the data was collected in the air at 298 K. The structures were solved by direct methods and refined by full-matrix least-squares based on *F*^2^ using SHELXTL programme package.^68^ All nonhydrogen atoms were found from the Fourier difference maps and refined anisotropically. However, the hydrogen atoms of organic ligands were generated geometrically and refined using a riding model. The hydrogen atoms of water molecules were located using Fourier difference maps. CCDC reference number 1865055–1865058, contains the supplementary crystallographic data for this paper.†

### *In situ* temperature-dependent PXRD of guest removal and inclusion

A bulk sample of LIFM-12 was ground, flattened in a sample plate and conducted with a Bruker D8 Advance diffractometer which was equipped with a high temperature chamber. Four sets of data were collected. (1) The sample was heated up to 308 K, kept for 1 hour, the PXRD pattern was collected in a nitrogen atmosphere. (2) The sample was continued to heat up to 435 K with a rate of 12 K min^−1^ in nitrogen, kept at 435 K for 1 hour and scanned in nitrogen. (3) The sample was later to cool to 298 K in natural rate in nitrogen, kept for 30 minutes, and scanned in nitrogen. (4) The samples were exposed to air, kept for 20 minutes, and then scanned in air.

### *In situ* temperature-dependent PXRD of C_3_H_8_ and CO_2_ filling

The bulk sample was prior to removing the guest of solvent to afforded LIFM-12, then ground, flatten in a sample plate, and conducted with a Bruker D8 Advance diffractometer which is equipped with a high temperature chamber. The sample was heated to 435 K under vacuum and kept for 3 hours, following by slowly cooling to 298 K. For C_3_H_8_ filling experiment, PXRD patterns of LIFM-12 were firstly collected under vacuum, then C_3_H_8_ was gradually filled into the chamber to the pressure of 300 mbar, keeping it for 30 min, then PXRD patterns were collected; then increase the pressure to 1 bar, keeping it for 30 min and PXRD patterns were collected. For CO_2_ filling experiment, the PXRD patterns of LIFM-12 was firstly collected under vacuum, then CO_2_ was replenished to the pressure of 500 mbar, keeping it for 30 min and PXRD patterns were collected again; then increase the pressure to 1 bar, keeping it for 30 min and PXRD patterns were collected.

### Gas adsorption

Gas adsorption isotherms for pressures in the range of 0–1.0 bar were obtained by a volumetric method using a Quantachrome autosorb-iQ2-MP gas adsorption analyzer. Argon adsorption isotherms were performed by the Belsorp-max gas adsorption analyzer. High pressure gas adsorption data was collected with the Belsorp-VC apparatus. The freshly prepared sample was transferred to a pre-dried and weighed analysis tube and evacuated at 433 K under a dynamic vacuum at 10^−3^ torr for 20 hours. Background adsorption values were deducted by blank measurement with an analysis tube. Gas adsorption measurements were performed using ultra-high purity Ar, N_2_, CO, O_2_, He, H_2_, CO_2_, CH_4_, C_2_H_6_, C_2_H_4_, C_3_H_8_, and C_3_H_6_ gas. All solvents used as sources of vapor are the grade of HPLC.

### Calculations of adsorption isosteric heats

The isosteric heats of CO_2_ adsorption for LIFM-12 were calculated base on the sorption data measured at 273, 298, 308 K by the virial fitting method, while that of H_2_ adsorption was calculated from the sorption data measured at 77, 87 K. A virial-type expression (eqn (1)) which is composed of parameters *a*_*i*_ and *b*_*i*_ is used. In eqn (1), *P* is the pressure in torr, *N* is the adsorbed amount in mmol g^−1^, *T* is the temperature in Kelvin, *a*_*i*_ and *b*_*i*_ are the virial coefficients which are independent of temperature, and *m* and *n* are the numbers of coefficients required to adequately describe the isotherms.1
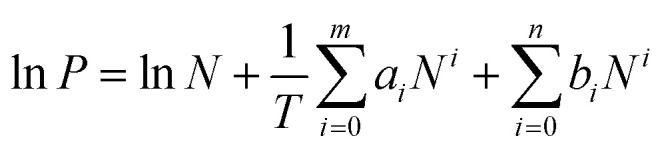


The values of the virial coefficients *a*_0_ through *a*_*m*_ were then applied to calculate the isosteric heat of adsorption (eqn (2)). In eqn (2), *Q*_st_ is the coverage-dependent isosteric heat of adsorption and *R* is the universal gas constant.2
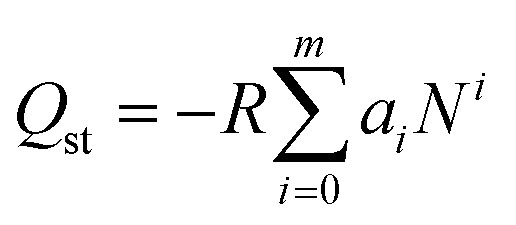


### Calculations of adsorption selectivity

Ideal adsorbed solution theory (IAST) developed by Myers and Prausnitz^69^ was used to calculate the selectivity of CO_2_/N_2_ (15 : 85), CO_2_/CO (50 : 50) and CO_2_/CH_4_ (50 : 50) mixture compositions in LIFM-12 from their respective single-component isotherms. The CO_2_, N_2_, CH_4_ isotherms were fitted to the single-site Langmuir equation and the CO isotherms to the dual-sites Langmuir equation. Selectivity was then calculated according to eqn (3), where *x*_*i*_ is the mole fraction of component *i* in the adsorbed phase and *y*_*i*_ is the mole fraction of component *i* in the bulk.3
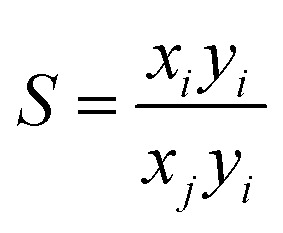


Another virial fitting method based on the following equation:

4 ln *N*/*P* = *A*_0_ + *A*_1_*N* + *A*_2_*N*^2^ + *A*_3_*N*^3^ +⋯

In eqn (4), *P* is pressure, *N* is amount adsorbed and *A*_0_, *A*_1_, *etc.* present virial coefficients. *A*_0_ is related to adsorbate–adsorbent interactions, and *A*_1_ describes adsorbate–adsorbate interactions. The Henry's law constant (*K*_H_) is equal to exp (*A*_0_).

The Henry's law selectivity for gas component *i* over *j* is calculated based on eqn (5):

5 *S*_*ij*_ = *K*_H*i*_ + *K*_H*j*_

### Simulation of surface area and pore size of LIFM-12

Simulated surface area and pore size were calculated with Materials Studio (Version 4.0, Accelrys, San Diego, CA) using a nitrogen-sized probe molecule (diameter 3.64 Å) and Argon-sized probe molecule (diameter 3.5 Å) with a grid interval of 0.40 Å and grid solution of “Fine”. The task of “Solvent surface” was chosen to calculate surface area and the “Connolly surface” was used for calculating pore size.

## Conflicts of interest

There are no conflicts to declare.

## Supplementary Material

RA-009-C9RA07301A-s001

RA-009-C9RA07301A-s002
