# Supplementary material for: Unusual adsorption behaviours and responsive structural dynamics via selective gate effects of an hourglass porous metal–organic framework
Source: RSC Adv. 2019 Nov 14;9(64):37222–31. doi: 10.1039/c9ra07301a (PMC9075530; doi:10.1039/c9ra07301a)
Supplement: RA-009-C9RA07301A-s001 [file RA-009-C9RA07301A-s001.pdf]

Electronic Supplementary Material (ESI) for RSC Advances.
This journal is © The Royal Society of Chemistry 2019

Electronic Supplementary Information

Exceptional Adsorption Behaviours and Responsive Structural Dynamics *via* Selective Gate Effects of an Hourglass Porous Metal-Organic Framework

Ying Xiong,[a,b] Yan-Zhong Fan, [a] Zhang-Wen Wei, [a] Cheng-Xia Chen,[a] Sha Chen,[a] Dawei Wang, [a] Mihail Barboiu,[a,c] Ji-Jun Jiang[a] and Cheng-Yong Su[a, d]*

[a] Lehn Institute of Functional Materials, School of Chemistry, Sun Yat-Sen University, Guangzhou 510275 (P.R. China)

[b] Life science institute, Jinzhou Medical University, Jinzhou 121001, (P.R. China)

[c] State Key Laboratory of Applied Organic Chemistry Lanzhou University, Lanzhou 730000, P.R. China.

S1. Presentation of crystal structures 2

S2. TGA Characterization 3

S3. Powder X-ray Diffraction 3

S4. The structural dynamics of LIFM-12 5

S5 Gas Selectivity, Adsorption Properties and Adsorption Isosteric Heat 8

S6 Single Crystal X-Ray Crystallography 10

S1. Presentation of crystal structures

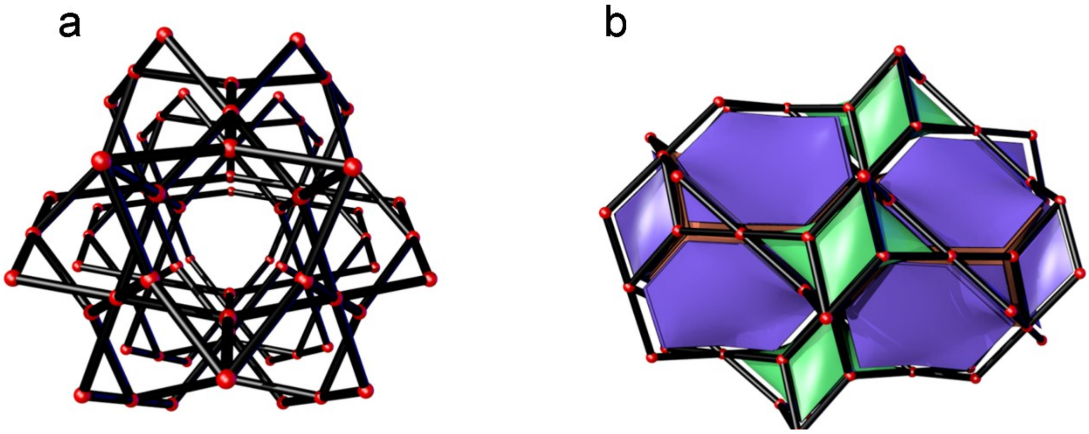

Figure S1. a, $ScD_{0.33}$ topology, and b, tiling of **LIFM-12**

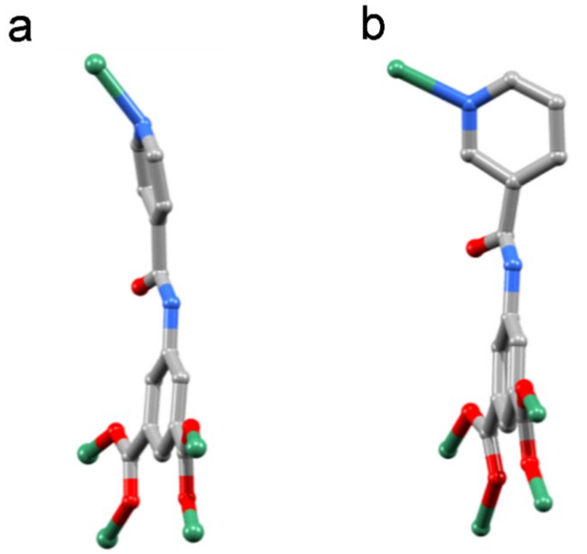

Figure S2. The curving plane of ligand of and coordination model of the ligand of **LIFM-12**

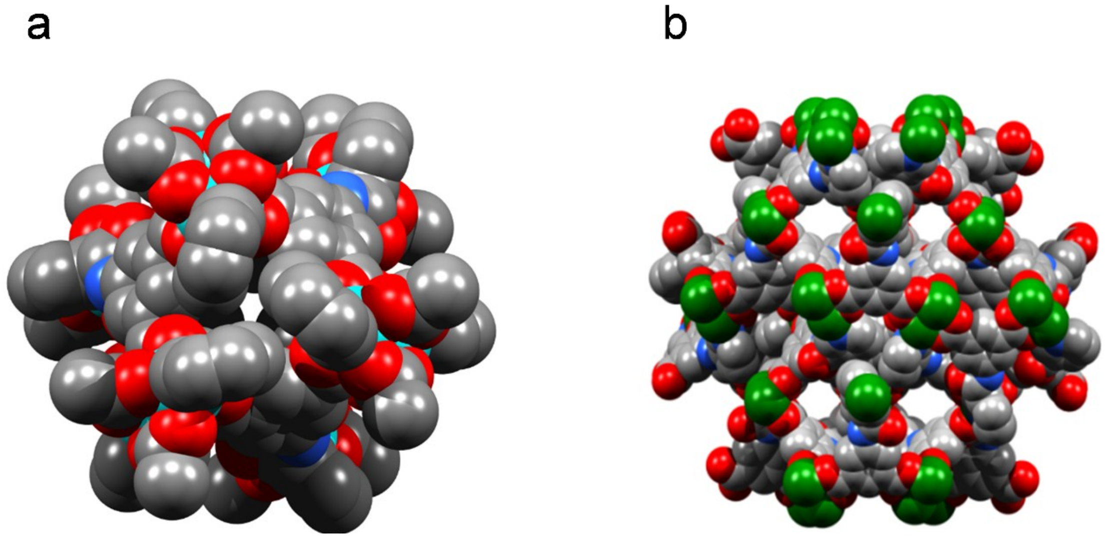

Figure S3. The view of narrow aperture from *c*-axis

S2. TGA Characterization

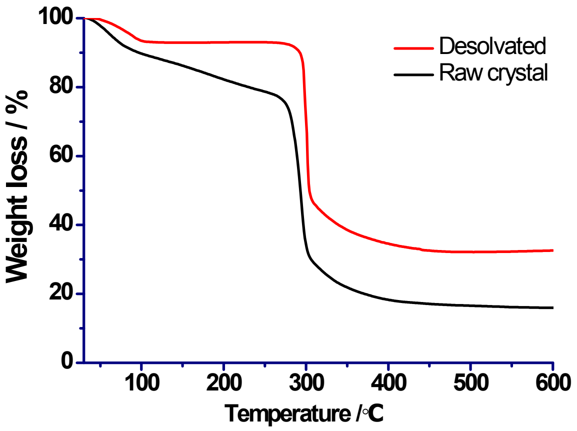

Figure S4. TGA curves of **LIFM-12** and **LIFM-12** in N_2. The step of **LIFM-12** curve before 100 °C can be arised as that water molecules were re-aborbed by the framework when the desolvated sample transferred to the TGA device

S3. Powder X-ray Diffraction

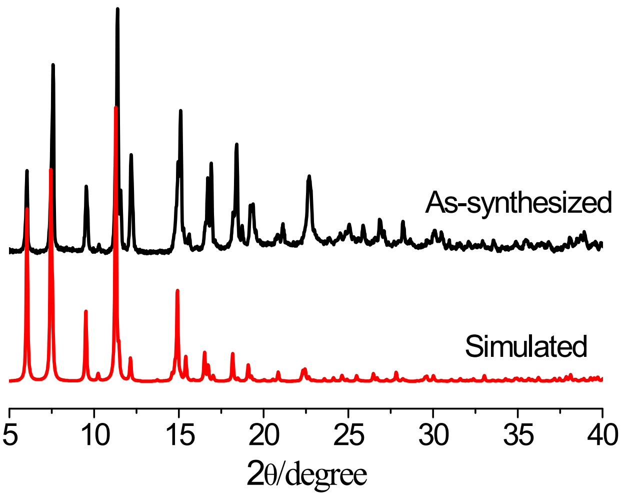

Figure S5. PXRD patterns of as-synthesized **LIFM-12** and simulated result

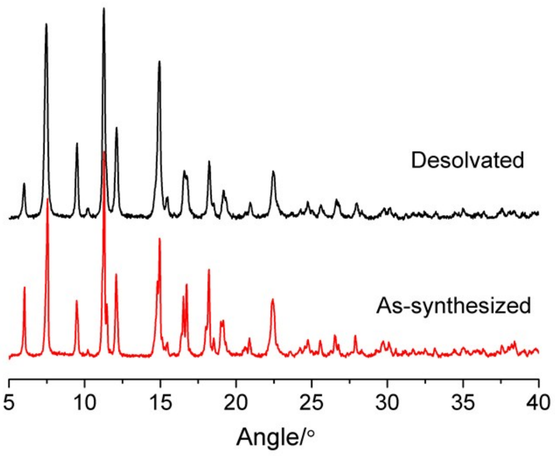

Figure S6. PXRD patterns of as-synthesized **LIFM-12** and its desolvated form, **LIFM-12**

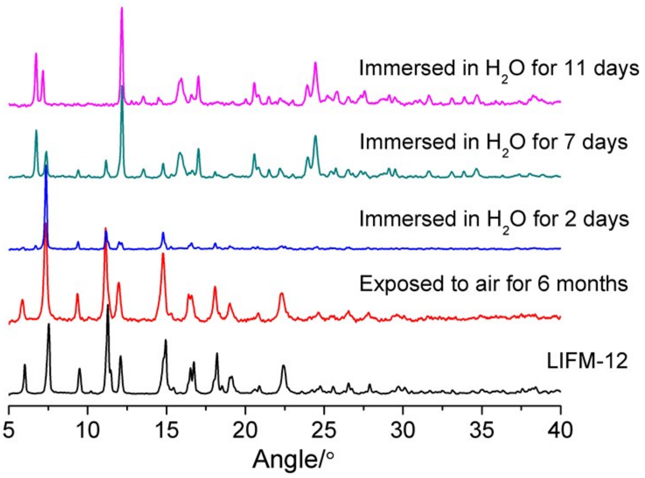

Figure S7. PXRD patterns of **LIFM-12** after exposed to air or immersed in H_2O

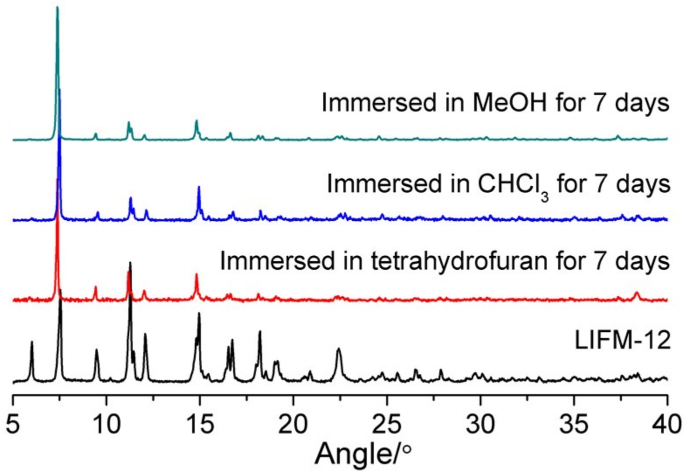

Figure S8. PXRD patterns of **LIFM-12** after immersed in organic solvents

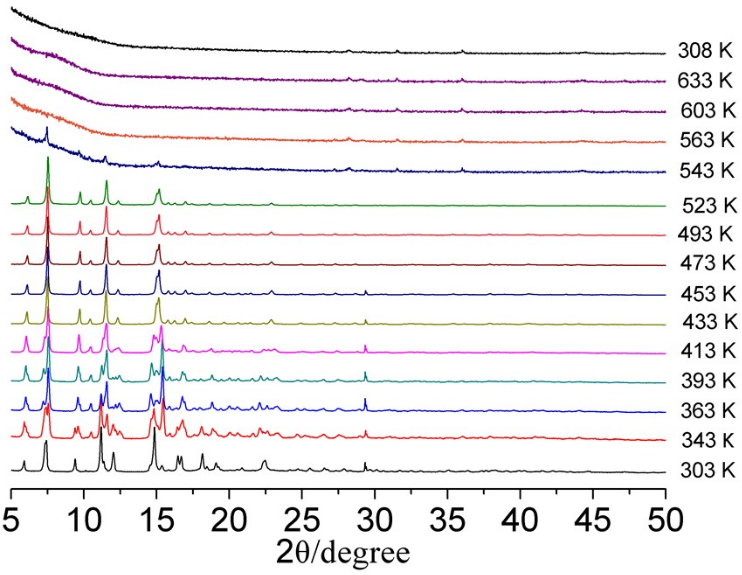

Figure S9 Full spectra of temperature-dependent PXRD patterns

S4 The structural dynamics of LIFM-12

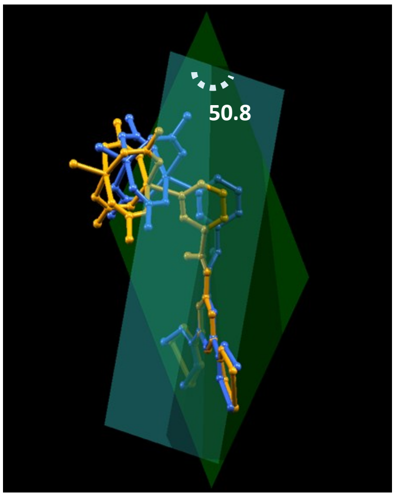

Figure S10 Structural comparing of **LIFM-12** and **LIFM-12-HT**. Yellow structure: **LIFM-12**. Blue structure **LIFM-12-HT**

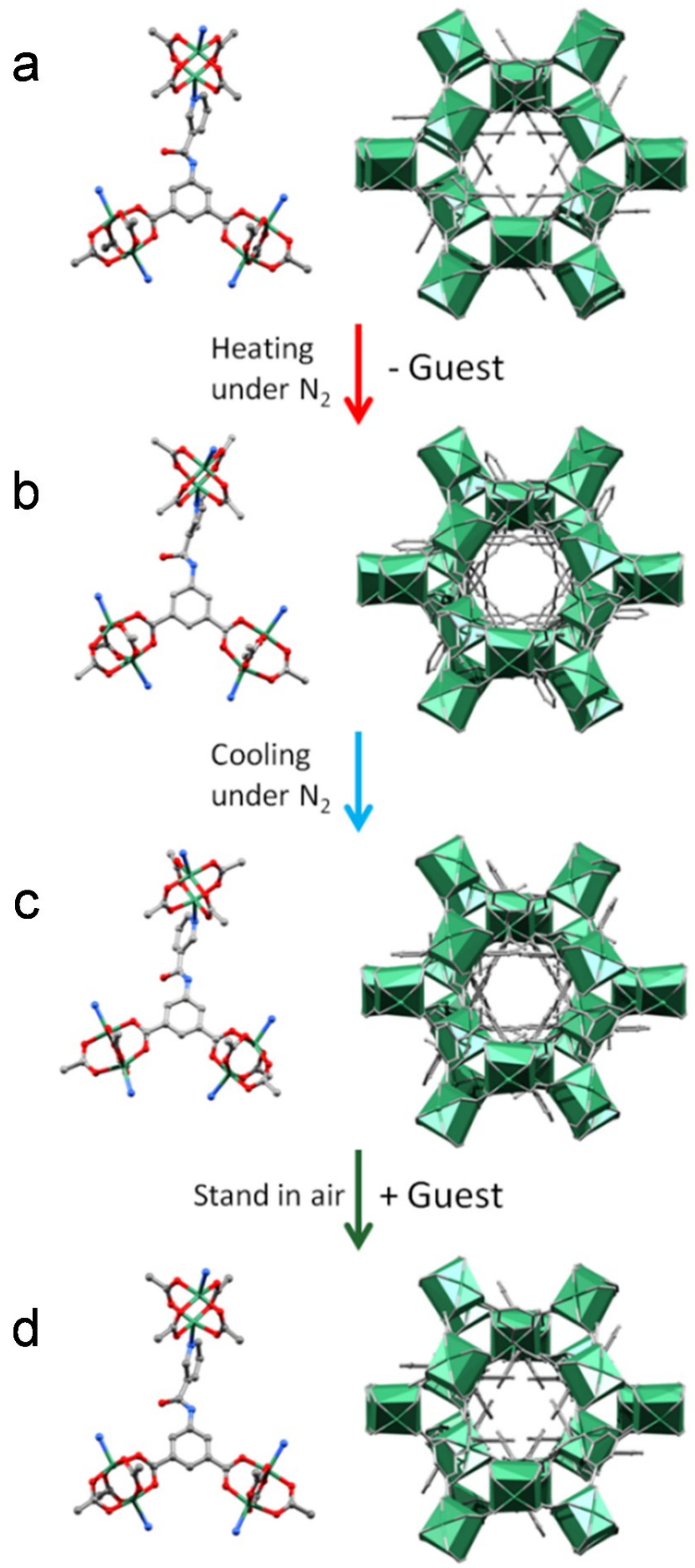

Figure S11 Scheme of *in situ* temperature-dependent PXRD patterns of guest removal and including

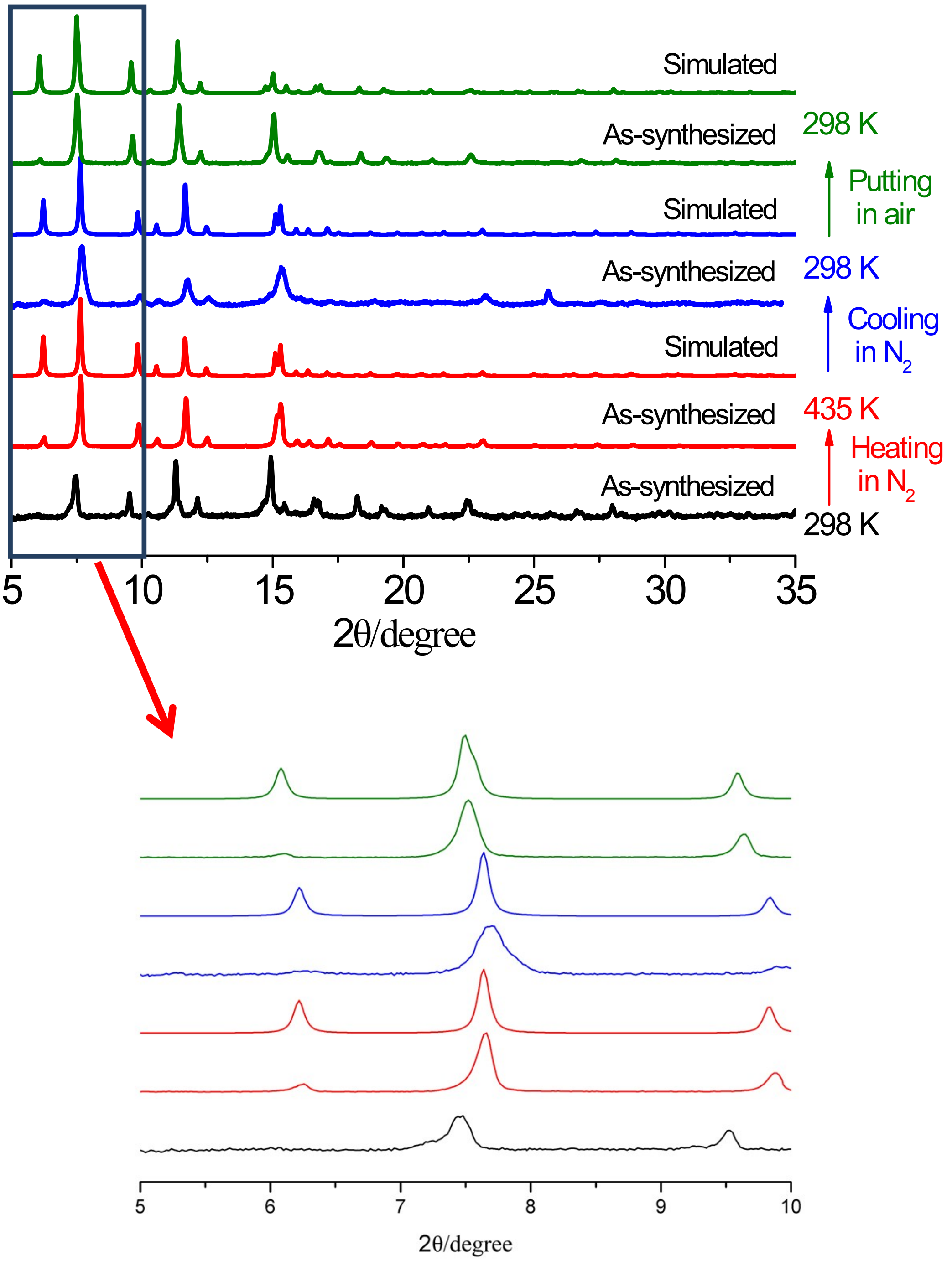

Figure S12 Simulated PXRD patterns from temperature-dependent X-ray single crystal studies of **LIFM-12** (black line), **LIFM-12-HT** (red line) , **LIFM-12-LT** (blue line), **LIFM-12-H_2O** (green line)

S5 Gas Selectivity, Adsorption Properties and Adsorption Isosteric Heat

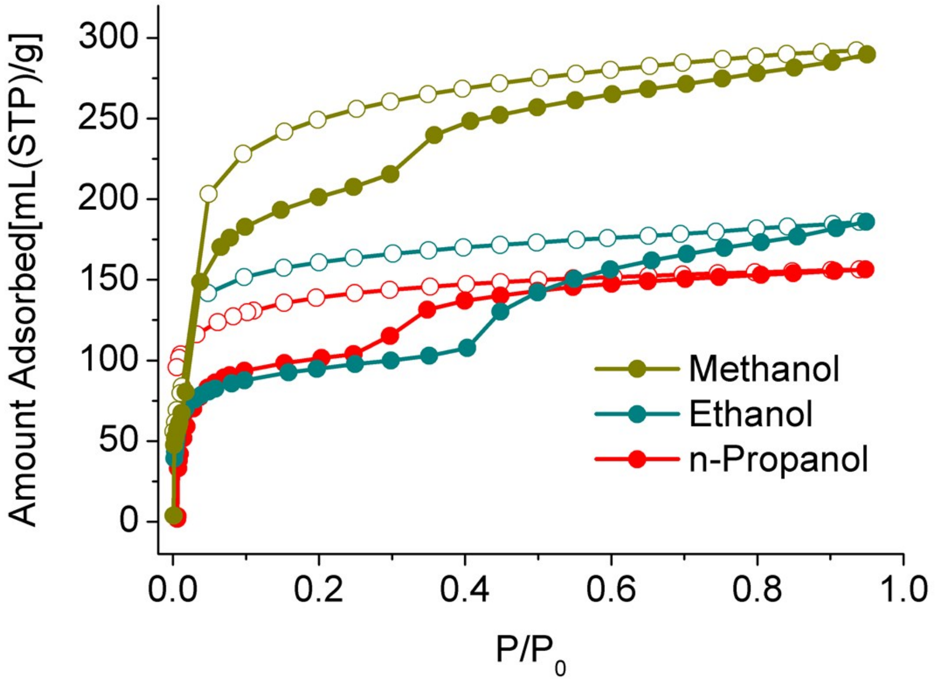

Figure S13 Vapor sorption isotherms of **LIFM-12**

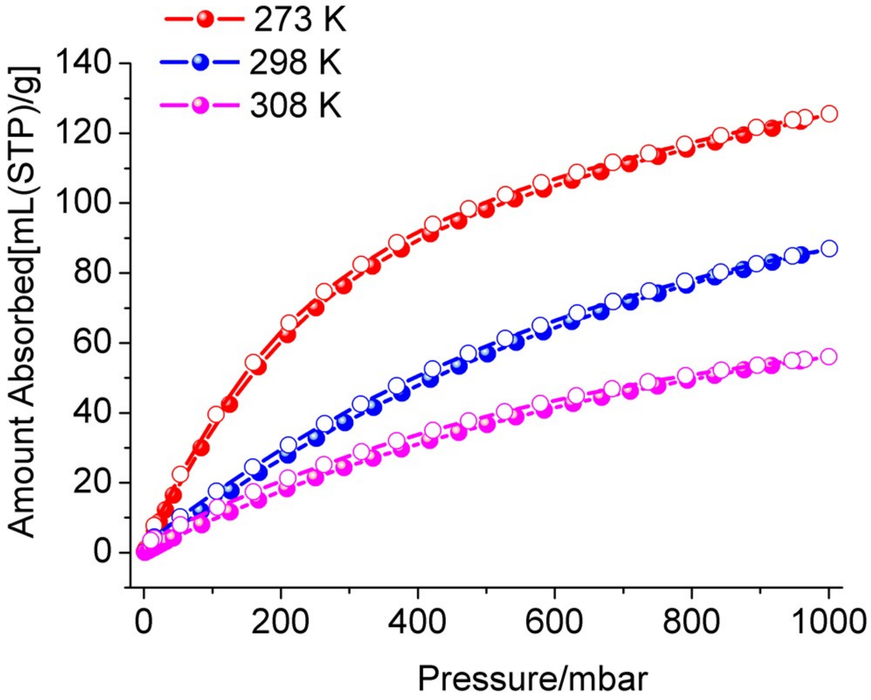

Figure S14 CO_2 sorption isotherms at 273 K, 298 K, 308 K of **LIFM-12**

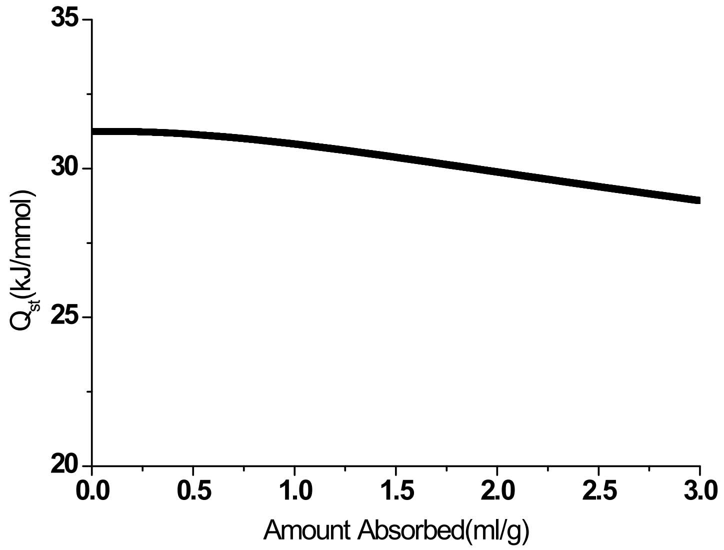

Figure S15 Isosteric heat of CO_2 adsorption of **LIFM-12**

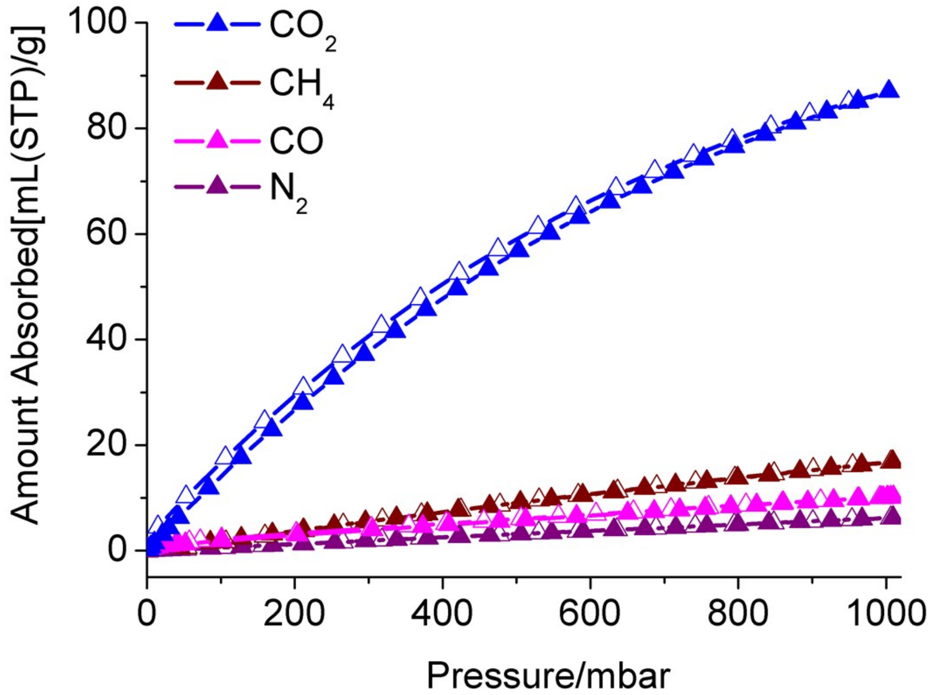

Figure S16 Gas sorption isotherms of **LIFM-12** at 298 K

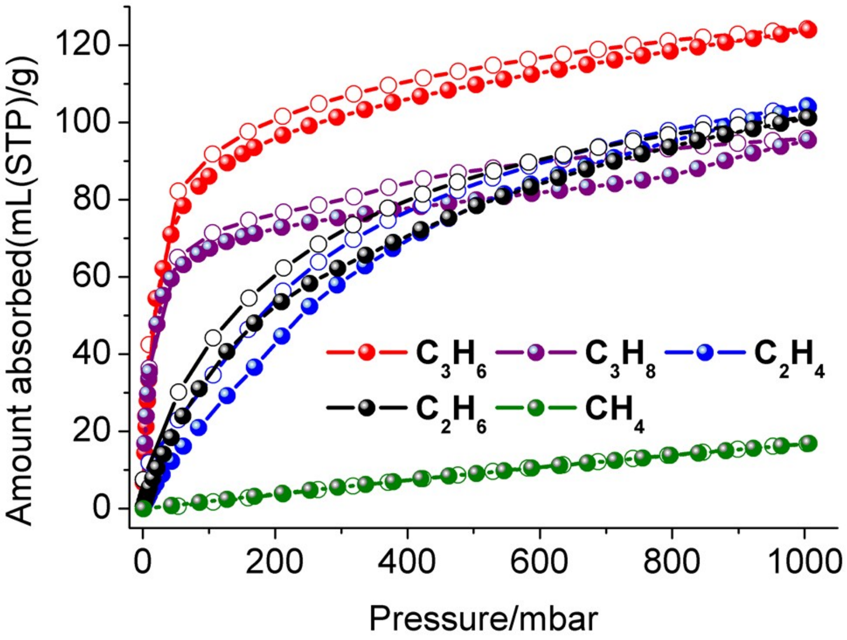

Figure S17 *n*-Alkanes sorption isotherms of **LIFM-12** at 298 K

S6. Single Crystal X-Ray Crystallography

Table S1. Crystallographic Data.

**Compound**	**LIFM-12**	**LIFM-12-HT**	**LIFM-12-LT**	**LIFM-12-H$_2$O**
**Formula**	C_{14} $CuN_2O_9H_8$	$C_{14}H_8N_2O_5Cu$	$C_{14}H_8N_2O_5Cu$	$C_{14}H_8N_2O_{6.5}Cu$
**Formula weight**	415.38	347.76	347.76	371.76
**Temperature(K)**	150 K	435 K	298 K	298 K
**Crystal system**	Hexagonal	Hexagonal	Hexagonal	Hexagonal
**Space group**	$R\bar{3}c$	$R\bar{3}c$	$R\bar{3}c$	$R\bar{3}c$
***a*(Å)**	18.3138(2)	17.993(3)	17.9678(16)	18.4309(18)
***c*(Å)**	69.7479(13)	69.267(14)	69.263(10)	70.011(16)
***V*(Å^3)**	20259.0(6)	19420(7)	19365(4)	20596(6)
**Z**	36	36	36	36
**Reflections collected**	17937	8927	8016	8429
**unique reflns**	3570	3058	2649	2909
***Dc* ((g·cm^{-3})**	1.226	1.070	1.074	1.056
**μ(mm^{-1})**	1.749	1.594	1.599	1.556
**GOF**	1.108	1.161	1.245	1.299
R_{int}	0.0237	0.1102	0.0741	0.0798
$R_1[I>2\sigma(I)]$	0.0787	0.1294	0.1312	0.1314
$wR_2[I>2\sigma(I)]$	0.2366	0.3374	0.3599	0.3386
**R_1(all data)**	0.0812	0.1962	0.1819	0.1628
**wR_2(all data)**	0.2405	0.3791	0.4013	0.3783
**Data**	0.99	0.98	0.95	0.97

completeness

Table S2 Selected bond length and angles.

Compounds	Bong length (Å)		Bond angle (°)	
LIFM-12	Cu1-O3	1.944(3)	O3-Cu1-O4	88.4(2)
	Cu1-O4	1.973(4)	O3-Cu1-O5	89.35(18)
	Cu1-O5	1.993(3)	O3-Cu1-N4	94.70(14)
	Cu1-O6	1.927(3)	O4-Cu1-O5	164.08(14)
	Cu1-N4	2.164(4)	O4-Cu1-N4	103.63(14)
			O5-Cu1-N4	92.27(13)
			O6-Cu1-O3	170.76(14)
			O6-Cu1-O4	89.42(18)
			O6-Cu1-O5	90.29(16)
			O6-Cu1-N4	94.54(13)

Symmetry code for LIFM-12: 1# '-Y,+X-Y,+Z'; 2# '+Y-X,-X,+Z'; 3# '+Y,+X,0.5-Z'; 4# '-X,-X+Y,0.5-Z'; 5# '-Y+X,-Y,0.5-Z'.

**LIFM-12-HT**	Cu1-O2	2.045(9)	O2-Cu1-N1	92.7(4)
	Cu1-O3	1.886(8)	O3-Cu1-O2	92.5(4)
	Cu1-O4	2.016(9)	O3-Cu1-O4	89.1(4)
	Cu1-O5	1.933(9)	O3-Cu1-O5	174.7(3)
	Cu1-N1	2.170(9)	O3-Cu1-N1	91.2(3)
			O4-Cu1-O2	155.3(4)
			O4-Cu1-N1	111.9(4)
			O5-Cu1-O2	88.3(4)
			O5-Cu1-O4	87.9(4)
			O5-Cu1-N1	94.0(4)

Symmetry code for LIFM-12-HT: 1# '-Y,+X-Y,+Z'; 2# '+Y-X,-X,+Z'; 3# '+Y,+X,0.5-Z'; 4# '-Y+X,-Y,0.5-Z'; 5# '-X,-X+Y,0.5-Z'.

**LIFM-12-LT**	Cu1-N1	2.176(9)	O1-Cu1-N1	92.5(3)
	Cu1-O1	2.025(9)	O2-Cu1-N1	91.0(3)

	Cu1-O2	1.891(8)	O2-Cu1-O1	91.6(4)
	Cu1-O3	1.930(9)	O2-Cu1-O3	175.0(3)
	Cu1-O4	1.999(9)	O2-Cu1-O4	90.2(4)
			O3-Cu1-N1	93.8(3)
			O3-Cu1-O1	89.4(4)
			O3-Cu1-O4	86.9(4)
			O4-Cu1-N1	111.1(3)
			O4-Cu1-O1	156.3(3)

Symmetry code for LIFM-12-LT: 1# '-Y,+X-Y,+Z'; 2# '+Y-X,-X,+Z'; 3# '+Y,+X,0.5-Z'; 4# '-Y+X,-Y,0.5-Z'; 5# '-X,-X+Y,0.5-Z'.

**LIFM-12-H_2O**	Cu1-O2	1.913(7)	O2-Cu1-O3	90.6(3)
	Cu1-O3	2.018(8)	O2-Cu1-O4	89.3(4)
	Cu1-O4	1.980(8)	O2-Cu1-O5	171.9(3)
	Cu1-O5	1.954(7)	O2-Cu1-N1	94(2)
	Cu1-N1	2.15(6)	O3-Cu1-N1	93.1(16)
			O4-Cu1-O3	162.5(3)
			O4-Cu1-N1	104.4(17)
			O5-Cu1-O3	89.3(4)
			O5-Cu1-O4	88.4(4)
			O5-Cu1-N1	94(2)

Symmetry code for LIFM-12-H_2O: 1# '-Y,+X-Y,+Z'; 2# '+Y-X,-X,+Z'; 3# '+Y,+X,0.5-Z'; 4# '-Y+X,-Y,0.5-Z'; 5# '-X,-X+Y,0.5-Z'.

Table S3 Measurement conditions and results of temperature-dependent X-ray single-crystal cycling test

	**Tempera-ture (K)**	**Atmos-phere**	**Formula**	**Space group**	**Unit cell (Å)**	**The solvent accessible volume**
**LIFM-12**	150	N_2	$[Cu(C_{14}N_2O_5H_8)]\cdot(C_3NOH_7)_{1.33}\cdot(H_2O)_2$	$R\bar{3}c$	a=18.3138 c=69.7479 V=20259.0	51.4%
**LIFM-12-HT**	435	N_2	$Cu(C_{14}H_7N_2O_5)$	$R\bar{3}c$	a=17.9928 c=69.2671 V=19420.3	45.7%
**LIFM-12-LT**	298	N_2	$Cu(C_{14}H_7N_2O_5)$	$R\bar{3}c$	a=17.9678 c=69.2632 V=19365.2	46.1%
**LIFM-12-H_2O**	298	Air	$(CuC_{14}H_7N_2O_5)\cdot 2H_2O$	$R\bar{3}c$	a=18.4309 c=70.0108 V=20596.3	52.7%

Table S4 BET surface areas calculated by Ar(87K) and N_2(77K) of **LIFM-12**

**Gas**	**Solvent surface($m^2\cdot g^{-1}$)**	**Pore volume($cc\cdot g^{-1}$)**
**Ar(87K)**	P/P_0=0.0063~0.038 c=3288.193 r=0.999997 S=950.068	0.50
**N_2(77K)**	P/P_0=0.069~0.089 c=126.117 r=0.999418 S=1221.102	0.55

Table S5 Summary of some MOFs with high CO_2 sorption amount at 1 bar*

MOFs	CO_2 uptake (mmol•g^{-1})	Reference
Mg-MOF-74	8.5	(S1,S2)
Cu(Me-4py-trz-ia)	8.3	(S3)
Co-MOF-74	7.5	(S1)
Zn-MOF-74(296 K)	7.3	(S1)
Ni-MOF-74	7.1	(S1)
Cu (bpy-1)$_2$ (SiF_6)	6.8	(S4)
Cu-TDPAT	5.9	(S5)
{[CuL]·DMF·2H_2O}$_n$**	5.0	(S6)
HKUST-1 (295 K)	4.7	(S7)
CuBTTri-mmen	4.1	(S8)
Bio-MOF-11	4.1	(S9)
$Zn_3(OH)(p\text{-}CDC)_{2.5}$	4.0	(S10)
LIFM-12	3.9	This work
$Zn_2(C_2O_4)(C_2N_4H_3)_2(H_2O)_{0.5}$	3.78	(S11)
MIL-53(Al)	2.6	(S12)

*Unless otherwise noted all data were obtained at 298 K. ** H_2L = 5-(4*H*-1,2,4-triazol-4-yl)benzene-1,3-dicarboxylic acid.

Table S6 CO_2/N_2, CO_2/CH_4, CO_2/CO Separation Selectivity of **LIFM-12.**

**Adsorbate**	**T/K**	**Calculated by viral method**				**Calculated by IAST method (Zero-converge)**
		K_H/mol g^{-1} torr^{-1}×10^{-3}	a_0/ln(mol g^{-1} torr^{-1})	b_0/ln(mol g^{-1} torr^{-1})	S_{ij}	S
**CO_2**	298	0.0671	-9.60967±0.00311	-0.10065±0.00293	—	—
**N_2**	298	0.00257	-12.87129±0.00653	1.09657±0.09888	26.1	23.2
**CH_4**	298	0.00863	-11.66003±0.00179	-0.16586±0.00627	7.8	7.9
**CO**	298	0.0643	-9.65208±0.05891	-33.88577±1.64267	1.0	1.1

Table S7 CH_4/C_2H_6, CH_4/C_2H_4, CH_4/C_3H_8 and CH_4/C_3H_6 Separation selectivity of **LIFM-12**

**Adsorbate**	**T/K**	**Calculated by viral method**				**Calculated by IAST method(Zero-converge)**
		K_H/mol g^{-1} torr^{-1}×10^{-3}	a_0/ln(mol g^{-1} torr^{-1})	b_0/ln(mol g^{-1} torr^{-1})	S_{ij}*	S
**CH_4**	298	0.00863	-11.66003±0.00179	-0.16586±0.00627	—	—
**C_2H_6**	298	0.168	-8.6911±0.02995	-0.33574±0.02766	19.47	15.2

**C_2H_4**	298	0.174	-8.65446±0.04365	-1.0293±0.12986	20.16	27.5
**C_3H_8**	298	3.49	-5.65659±0.02376	-0.50771±0.02197	456.55	86.8
**C_3H_6**	298	2.50	-5.99337±0.02823	-0.29911±0.02774	289.69	273.5

*The Henry's law selectivity for gas component i over CH_4 at the speculated temperature is calculated based on equation: $S_{ij}=K_H(i)/K_H(CH_4)$

Reference

1. A. Ö. Yazaydın, R. Q. Snurr, T.-H. Park, K. Koh, J. Liu, M. D. LeVan, A. I. Benin, P. Jakubczak, M. Lanuza, D. B. Galloway, J. J. Low and R. R. Willis, *Journal of the American Chemical Society*, 2009, **131**, 18198-18199.
2. S. R. Caskey, A. G. Wong-Foy and A. J. Matzger, *Journal of the American Chemical Society*, 2008, **130**, 10870-10871.
3. D. Lässig, J. Lincke, J. Moellmer, C. Reichenbach, A. Moeller, R. Gläser, G. Kalies, K. A. Cychosz, M. Thommes, R. Staudt and H. Krautscheid, *Angewandte Chemie International Edition*, 2011, **50**, 10344-10348.
4. S. D. Burd, S. Ma, J. A. Perman, B. J. Sikora, R. Q. Snurr, P. K. Thallapally, J. Tian, L. Wojtas and M. J. Zaworotko, *Journal of the American Chemical Society*, 2012, **134**, 3663-3666.
5. B. Li, Z. Zhang, Y. Li, K. Yao, Y. Zhu, Z. Deng, F. Yang, X. Zhou, G. Li, H. Wu, N. Nijem, Y. J. Chabal, Z. Lai, Y. Han, Z. Shi, S. Feng and J. Li, 2012, **51**, 1412-1415.
6. L. Wen, W. Shi, X. Chen, H. Li and P. Cheng, 2012, **2012**, 3562-3568.
7. Q. Min Wang, D. Shen, M. Bülow, M. Ling Lau, S. Deng, F. R. Fitch, N. O. Lemcoff and J. Semanscin, *Microporous and Mesoporous Materials*, 2002, **55**, 217-230.
8. T. M. McDonald, D. M. D'Alessandro, R. Krishna and J. R. Long, *Chemical Science*, 2011, **2**, 2022-2028.
9. J. An, S. J. Geib and N. L. Rosi, *Journal of the American Chemical Society*, 2010, **132**, 38-39.
10. Y.-S. Bae, O. K. Farha, A. M. Spokoyny, C. A. Mirkin, J. T. Hupp and R. Q. Snurr, *Chemical Communications*, 2008, DOI: 10.1039/B805785K, 4135-4137.
11. R. Vaidhyanathan, S. S. Iremonger, K. W. Dawson and G. K. H. Shimizu, *Chemical Communications*, 2009, 5230-5232.
12. B. Arstad, H. Fjellvåg, K. O. Kongshaug, O. Swang and R. J. A. Blom, 2008, **14**, 755-762.
